# Synthesis and 5α-Reductase Inhibitory Activity of C_21_ Steroids Having 1,4-diene or 4,6-diene 20-ones and 4-Azasteroid 20-Oximes

**DOI:** 10.3390/molecules17010355

**Published:** 2011-12-30

**Authors:** Sujeong Kim, Yong-ung Kim, Eunsook Ma

**Affiliations:** 1 College of Pharmacy, Catholic University of Daegu, Hayang, 712-702, Korea; 2 College of Herbal Bio-industry, Daegu Hany University, Gyeongsangbuk-do 712-715, Korea

**Keywords:** 5α-reductase inhibitor, BPH, 4-azasteroid-20-oxime

## Abstract

The synthesis and evaluation of 5α-reductase inhibitory activity of some 4-azasteroid-20-ones and 20-oximes and 3β-hydroxy-, 3β-acetoxy-, or epoxy-substituted C_21_ steroidal 20-ones and 20-oximes having double bonds in the A and/or B ring are described. Inhibitory activity of synthesized compounds was assessed using 5α-reductase enzyme and [1,2,6,7-^3^H]testosterone as substrate. All synthesized compounds were less active than finasteride (IC_50_: 1.2 nM). Three 4-azasteroid-2-oximes (compounds **4**, **6** and **8**) showed good inhibitory activity (IC_50_: 26, 10 and 11 nM) and were more active than corresponding 4-azasteroid 20-ones (compounds **3**, **5** and **7**). 3β-Hydroxy-, 3β-acetoxy- and 1α,2α-, 5α,6α- or 6α,7α-epoxysteroid-20-one and -20-oxime derivatives having double bonds in the A and/or B ring showed no inhibition of 5α-reductase enzyme.

## 1. Introduction

It has long been established that prostatic growth is stimulated by androgens and more recent investigations suggest that this tropic support is provided by 5α-dihydrotestosterone (DHT), rather than by the classic testicular hormone testosterone (T) [1]. Metabolically DHT is made from T by the action of the enzyme steroid 5α-reductase (5AR). This enzyme also catalyzes the NADPH-dependent reduction of the ∆^4^ double bond of several other steroid substrates [2,3]. A deficiency of 5AR in males results in an incomplete differentiation of external genitalia at birth [4]. On the other hand, abnormally high 5AR activity in humans results in excessively high DHT levels in peripheral tissues, which is implicated in the pathogenesis of prostate cancer, benign prostatic hyperplasia (BPH), acne and male pattern baldness [5,6]. It is presently recognized that there are two genes encoding two distinct isozymes of 5AR that are differentially expressed in human tissues and referred to as type 1 5AR (5AR1) and type 2 5AR (5AR2) [7,8,9]. Although 5AR1 is predominantly expressed in the skin and liver, 5AR2 is mainly expressed in prostate, seminal vesicles, liver and epididymis [10].

**Figure 1:**
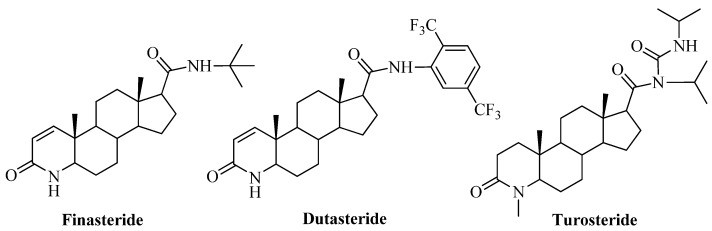
Representative 5α-reductase inhibitors.

Various steroidal [10,11,12] and non-steroidal [13,14,15] inhibitors have been synthesized and tested against 5AR. Of these, finasteride (PROSCAR^®^, Figure 1), a type II-selective 5α-reductase inhibitor, was the first 5AR inhibitor approved in the USA for the treatment of BPH and prostate cancer. It has also been demonstrated that dutasteride (Figure 1) acts as a 5AR1 and 5AR2 inhibitor [16] and turosteride (Figure 1) is selective for the type II isoform of 5α-reductase. However, since finasteride is slow acting and produces side effects affecting sexual function [17], this has caused us and others to seek new classes of steroidal and non-steroidal inhibitors.

The steroidal pharmacophore provides as an anchor between the key A-ring lactam amide and C-17 substitutent while the former acts as an enzyme transition state mimic of intermediate enolate, the latter significantly enhances potency via binding a pocket largely lipophilic in nature [18]. On the other side, the introduction of polar groups in C-17 is proved to modulate and modify the activity of these testosterone-derived inhibitors [19]. Taking into consideration that β-substitution at C-17 could dramatically affect the potency, a large number of modifications were carried out to find inhibitors [18]. The stereoselective synthesis [20] of mono- and dialkylcarboxamides as 17-β side chains were reported to enhance inhibiting activity [21]. 

Of reported C_21_ steroid derivatives, some steroidal 20-hydroxyimino derivatives were reported as dual inhibitors of CYP 17 (17α-hydroxylase/C17,20-lyase) and 5α-reductase [10,22]. Recently, 3- and 19-oximes of 16α,17α-cyclohexanoprogesterone derivatives were synthesized and evaluated to probe the surfaces of progesterone receptor’s and two other protein ligand binding pockets neighboring to 3- and 19-positions of steroid core [23]. Furthermore, 3-acetoxy-5α,6α-epoxyprogn-16-en-20-one, which doesn’t have a double bond in the A or B ring [18] and 6α,7α-epoxy progesterone [11] were reported to exhibit higher 5AR inhibitory activity than finasteride. Furthermore, steroidal epoxy compounds have been used as important intermediates to synthesize potent inhibitors of 5AR, since their facile ring opening allows the introduction of various functionalities in a stereospecific manner. Also, it was reported that the 5AR inhibitory activity was tightly dependent on the presence, position, and number of unsaturations on the A-C rings of steroid skeleton [24].

In order to evaluate the combined effect of the hydroxyimino group, epoxy ring, and additional double bond on the 5AR inhibitory activity of the steroidal compound, on the basis of the facts described in these references, we report here the synthesis and 5AR inhibitory activity of some 4-azasteroids bearing a hydroxyimino group at C-20 and 3-hydroxy- or 3-acetoxy-, or epoxy-substituted C_21_ steroidal 20-one and 20-oxime derivatives having double bond in the A or B ring. 

## 2. Results and Discussion

### 2.1. Chemistry

**Scheme 1:**
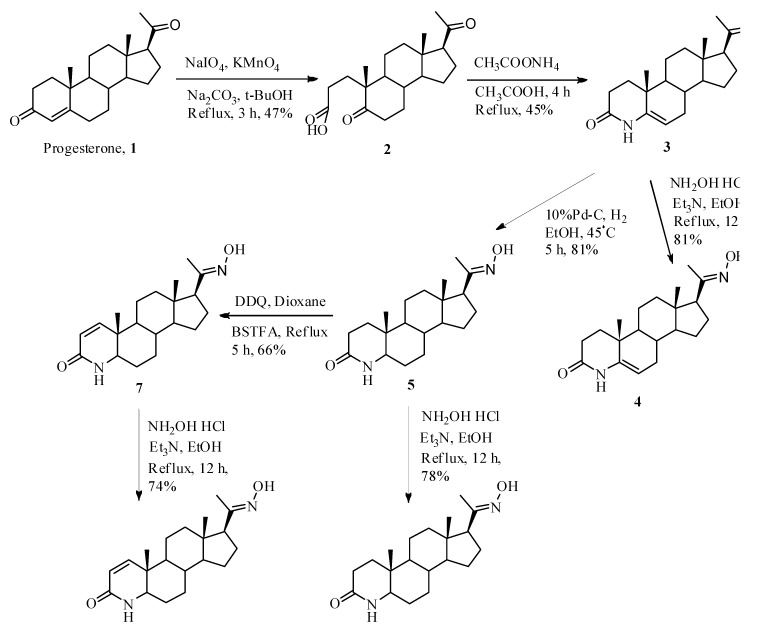
Synthesis of 4-azasteroid 20-oxime derivatives.

**Scheme 2:**
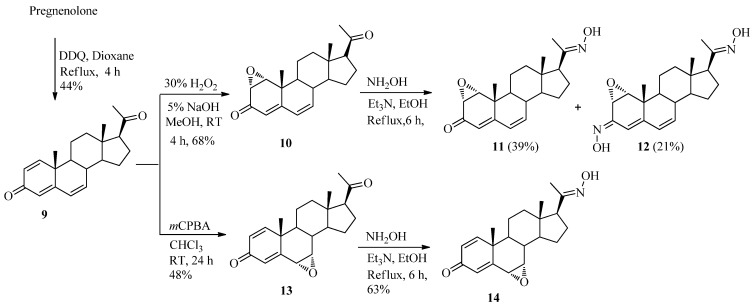
Synthesis of epoxypregnadiene-3,20-dione-20-oximes.

The syntheses of 4-azapregnene or 4-azapregnane 20-oxime and pregnane or 5-pregnene 20-oxime derivatives are shown in Scheme 1 and Scheme 2. Progesterone (**1**) was treated with sodium periodate and potassium permanganate to form 5,20-dioxo-A-nor-3,5-secopregnane-3-oic acid (**2**) [25]. This compound was treated with ammonium formate to afford 4-aza-5-pregnene-3,20-dione (**3**) in 45% yield, which was converted into its 20-oxime analog **4** in 71% yield using hydroxylamine. The 20-C=O carbon peak (209.4 ppm) of compound **3** was not observed in ^13^C-NMR spectrum of compound **4**, but the 20-C=NOH carbon peak was identified at 158.9 ppm. In the IR spectrum of **4**, the characteristic oxime bands were observed at 3,575 cm^−1 ^(OH), 1,668 cm^−1^ (C=N) and 953 cm^−1^ (N-O), and N-H and 3-C=O bands of A ring of 4-azasteroid skeleton were observed at 3,200 and 1,660 cm^−1^, respectively.

To assign the configuration of the 20-oxime, an analysis of its COSY spectrum were used. The COSY spectrum of 20-oxime **4** did not exhibit correlation peaks of the H-16 and the proton 20-oxime group, consequently, 20-oxime **4** was identified as the *E*-isomer.

The catalytic hydrogenation reaction of **3** with 10% Pd-C in ethanol afforded 4-aza-pregnane-3,20-dione (**5**) in 81% yield, which was refluxed with DDQ in the presence of BSTFA to give the 1,2-position dehydrogenated compound, 4-aza-1-pregnene-3,20-dione (**7**) in 66% yield. The ^1^H-NMR spectrum of **7** showed peaks at δ 6.80 (H-1) and 5.82 (H-2) ppm for two double bond hydrogens. Two carbonyl carbons and double bond carbons were assigned at 209.2 (C-20), 166.5 (C-3), 150.9 (C-1) and 123.0 (C-2) ppm in the ^13^C-NMR spectrum. The compounds **5** and **7** were treated with hydroxylamine to the synthesize 20-oxime analogs **6** and **8** and the structures of **6** and **8** were identified by same method described for compound **4**.

The syntheses of 1,2-epoxy- or 6,7-epoxy-pregnadiene or pregnatriene derivatives were shown in Scheme 2. Pregnenolone was refluxed with four equivalents of DDQ in dioxane to form 1,4,6-pregnatriene-3,20-dione (**9**). This trienone was treated with 30% hydrogen peroxide under basic conditions to synthesize 1α,2α-epoxy-4,6-pregnadiene-3,20-dione (**10**) and the reaction of **9** and *m*-chloroperoxybenzoic acid (*m*CPBA) at room temperature afforded 6α,7α-epoxy-1,4-pregnadiene-3,20-dione (**13**), stereoselectively and regioselectively. According to the same manner reported [26], the stereochemistry of α-epoxidation of compound **10** was confirmed by irradiation of H-1 (3.47 ppm) which showed a NOE to C-19 methyl proton (1.19 ppm) in 1D-NOESY spectrum and α-epoxidation of compound **13 **was also confirmed by irradiation of H-6 (3.37 ppm) which showed a NOE to C-19 methyl proton (1.21 ppm) in the 1D-NOESY spectrum.

**Figure 2:**
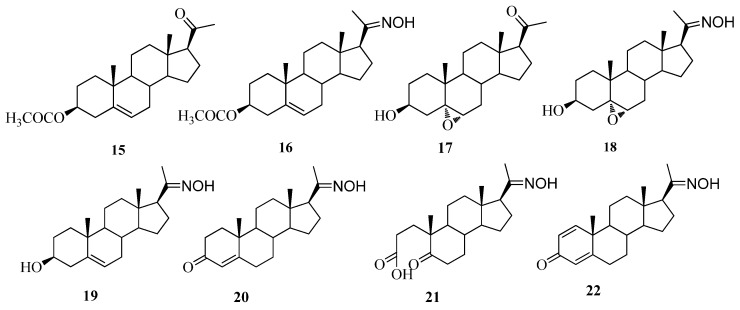
Previous synthesized compounds [29].

The reaction of **10** and hydroxylamine under reflux conditions for 6 h gave 20-oxime **11** and 3,20-dioxime **12**. The two oxime hydrogen peaks (H-3 and H-20) of compound **12** were identified at 11.34 and 10.39 ppm in its ^1^H-NMR spectrum and the two carbon peaks (C-3 and C-20) were identified at 147.3 and 149.5 ppm in the ^13^C-NMR spectrum. The structure of **11** was identified by observing one carbonyl carbon peak (C-3) at 183.2 ppm in the ^13^C-NMR spectrum and one oxime hydrogen peak at 10.28 ppm in the ^1^H-NMR spectrum. The configuration of 20-oxime **12** was identified as *E*- by observing no correlation peak of the H-16 and the proton 20-oxime group in the COSY spectrum. The 3-oxime **12** showed no correlation peak of the H-4 or H-2 and the proton 3-oxime group in the COSY spectrum. To support the configuration of the 3-oxime, it should be noted that the suggestions about anisotropic influence of N-O bond in *E*- an *Z*-isomeric 3-steroidal oxime on chemical shifts differences of 4-H signal were experimentally confirmed in this study [27,28]. The chemical shift of H-4 peak (6.15 ppm) of **12** was shifted further downfield than H-4 peak (5.87 ppm) of **11**, thus confirming the *E*-configuration of the 3-oxime group. Compound **13** was reacted with hydroxylamine under reflux conditions for 6 h to yield 6α,7α-epoxypregna-1,4-diene-3,20-dione-20-oxime (**14**). One carbonyl carbon peak (C-3) of **14** at 185.4 ppm was observed in the corresponding ^13^C-NMR spectrum. The structures of compounds **15**–**22** are shown in Figure 2.

### 2.2. Testosterone 5α-Reductase Assay

The series of C_21_ steroids **1–22** and finasteride as a reference were evaluated for their inhibitory activity against testosterone 5α-reductase prepared from rat prostates. It has been known that both the type 1 and the type 2 isozymes are present in the ventral prostate of the rat [8]. Since the rat prostate extracts for measuring testosterone 5α-reductase inhibition were used in a neutral pH buffer, both isozymes were assayed in this study due to differences in its biochemical properties. The inhibition (IC_50_ values or % inhibition at constant concentration for the less active compounds) determined against rat prostatic testosterone 5α-reductase are summarized in Table 1.

To compare the inhibitory activity, progesterone (**1**) was evaluated and showed the potent inhibitory activity with IC_50_ value of 6.5 nM in our assay. Compound **2** exhibited only a weak inhibitory activity toward the enzyme, which indicated the presence of steroid ring A is important for the inhibitory activity. The introduction of lactam ring in steroid ring A showed a broad range of inhibitory activities from moderate inhibition for compound **3** to potent inhibition for compounds **5** and **7**. The 4-azasteroid **5**, the lactam analogue of progesterone, was as active as progesterone, indicating that the replacement of sp^2^ carbon atoms at C-4 and C-5 position into sp^3^ nitrogen and sp^3^ carbon atom at C-4 and C-5 position, respectively, has little effect on the activity. The presence of a double bond between C-5 and C-6, as shown in compound **3**, significantly reduced the activity, while the introduction of a double bond between C-1 and C-2, as shown in compound **7**, led to a moderate decrease in the activity. The 20-oxime analogues (compounds **4**, **6** and **8**) showed potent inhibition, indicating that the replacement of oxygen atom on C-20 with an oxime functional group has a positive effect on the activity. The 20-oxime analogue of compounds **3** and **5**, as exemplified by compounds **4** and **6**, showed a substantial increase in activity, while the 20-oxime analogue of compound **7**, as shown in compound **8**, almost did not affect the activity. The 17-hydroxyimino analog **8 **(IC_50_: 11 nM), which has same steroidal skeleton as finasteride, except for the substituent at the C-17 position, showed 9.2 times less inhibitory activity than finasteride (IC_50_: 1.2 nM). This result again confirms that the presence of a lipophilic group at C-17 is very important.

1,4,6-Pregnatriene-3,20-dione (**9**) showed moderate inhibition, indicating that the introduction of additional double bonds at 1,2- and 6,7-position of progesterone resulted in the diminished activity compared to the progesterone. While it was already reported that a conjugated system of C-3, C-4, and C-5 position showed good activity [30,31], our result showed bad activity. The epoxy compounds **10** and **13** showed no inhibitory activity, indicating that the presence of hydrophilic epoxy moiety in 1,2- or 6,7-position results in a loss of activity. 

**Table 1:** Screening results of testosterone 5α-reductase inhibitory activity.

Compound No.	IC_50_ (M)	Compound No.	IC_50_ (M)
**1**	6.5 × 10^−9^	**13**	>1 × 10^−5^
**2**	>1 × 10^−5^	**15**	>1 × 10^−5^
**3**	2.3 × 10^−6^	**16**	>1 × 10^−5^
**4**	2.6 × 10^−8^	**17**	>1 × 10^−5^
**5**	6.1 × 10^−6^	**18**	>1 × 10^−5^
**6**	1.0 × 10^−8^	**19**	>1 × 10^−5^
**7**	1.4 × 10^−8^	**20**	1.4 × 10^−8^
**8**	1.1 × 10^−8^	**21**	2.1 × 10^−6^
**9**	7.6 × 10^−7^	**22**	1.4 × 10^−7^
**10**	>1 × 10^−5^	**Finasteride**	1.2 × 10^−9^

The experiments were done in triplicate.

3β-Acetoxypregnenolone (**15**) and 20-oxime derivative (**16**), 3β-hydroxy-5α,6α-epoxypregnane and 20-oxime (**17** and **18**), 3β-hydroxy pregn-4-ene-20-oxime (**19**) exhibited no inhibition for 5α-reductase enzyme. This result could indicate that the carbonyl group directly bonded at C-3 position is critical for the inhibitory activity. Compound **20**, the 20-oxime analogue of progesterone (**1**), was less active than corresponding carbonyl compound. 1,4-Pregnadiene-20-oxime (**22**) exhibited inhibitory activity similar to that of compound **9** and was more active than 6α,7α-epoxy-1,4-pregnadiene-20-one (**13**) having an epoxy ring at the 6,7 position. 1,2- or 6,7-Epoxy rings and a double bond at C-1-C-2 in addition to C-4 seems to be detrimental to the activity. 

## 3. Experimental

### 3.1. General Methods

All non-aqueous reactions were performed under a dry atmosphere of nitrogen. The commercial reagents were purchased from Aldrich, Fluka, or Sigma. Solvents were purified and dried prior to use. Melting points were measured on Thomas-Hoover melting point apparatus and not corrected. ^1^H-, ^13^C- NMR, HSQC, HMQC and NOESY spectra were taken on a Varian 400 MHz spectrometer in CDCl_3 _and DMSO-*d_6_*. Chemical shifts (δ) are in parts per million (ppm) relative to tetramethylsilane, and coupling constants (*J*) are in Hertz. IR spectra were determined on FT-IR JASCO 4100 spectrometer. GC/MS spectra were obtained on a Shimadzu QP 5050 and JEOL GC Mate 2 mass spectrometers. Analytical TLC was performed on pre-coated silica gel 60 F_254_ plates (Merck). Solvent systems for TLC were ethyl acetate/*n*-hexane mixtures and 10% MeOH in dichloromethane. Column chromatography was carried out on Merck silica gel 9385 (230–400 mesh) eluting with ethyl acetate/*n*-hexane or methanol/dichloromethane mixtures.

### 3.2. Synthesis

The synthesis of steroids **2**–**14** (Schemes 1 and Scheme 2) was briefly described below and the preparation of the compounds **15**–**22** (Figure 2) was previously reported [29]. 

#### 3.2.1. 5,20-Dioxo-A-nor-3,5-secopregnane-3-oic acid (**2**)

To a solution of 4-pregnene-3,20-dione (**1**, 2 g, 6.36 mmol) in *tert*-butanol (20 mL), anhydrous sodium carbonate (1.1 g, 10.13 mmol) and H_2_O (2 mL) were added and the reaction mixture was refluxed. A solution of sodium periodate (11.4 g, 53.25 mmol) and potassium permanganate (140 mg, 0.89 mmol) in H_2_O (30 mL) was dropwise added to refluxed reaction mixture that was refluxed for an additional 3 h. The precipitate was filtered, washed with water and the filtrate was acidified with 5M HCl and extracted with dichloromethane. The organic phase was dried with anhydrous MgSO_4_, filtered, eliminated in vacuum. The crude product was purified by silica gel column chromatography (ethyl acetate: *n*-hexane = 1:3) to give **2** as a pale yellow solid. Yield: 1 g (47%), m.p.: 176–178 °C; ^1^H-NMR (CDCl_3_) δ: 2.13 (3H, s, 21-CH_3_), 1.13 (3H, s, 19-CH_3_), 0.69 (3H, s, 18-CH_3_); ^13^C-NMR (CDCl_3_) δ: 214.8 (C-20), 209.5 (C-5), 179.0 (COOH), 63.6, 56.1, 50.6, 48.1, 44.2, 38.6, 38.2, 35.0, 31.7, 31.4, 29.4, 29.2, 24.6, 23.0, 21.7, 20.5, 13.6; GC-MS (EI) *m/z*: 334 [M]^+^, 316 [M-H_2_O]^+^, 262 [M-CH_2_CH_2_COO]^+^; IR, cm^−1^: 3148 (OH), 1734 (20-C=O), 1698 (3-C=O and COO).

#### 3.2.2. 4-Aza-5-pregnene-3,20-dione (**3**)

To a mixture of 5,20-dioxo-A-*nor*-3,5-secopregnane-3-oic acid (**2**, 200 mg, 0.60 mmol) in acetic acid (2 mL) was added ammonium acetate (138 mg, 1.80 mmol) and refluxed for 4 h. The reaction mixture was evaporated to remove acetic acid and washed with water, neutralized with saturated NaHCO_3_ and extracted with dichloromethane. The organic phase was dried with anhydrous MgSO_4_, filtered, and eliminated under vacuum. The crude product was purified by silica gel column chromatography (methanol-dichloromethane = 1:49) to give **3** as a pale yellow solid. Yield : 85 mg (45%), m.p.: 158–160 °C; ^1^H-NMR (CDCl_3_) δ: 7.34 (1H, s, NH), 4.81 (1H, t, *J* = 2.8 Hz, H-6), 2.55 (1H, t, *J* = 9.0 Hz, H-17), 2.14 (3H, s, 21-CH_3_), 1.10 (3H, s, 19-CH_3_), 0.66 (3H, s, 18-CH_3_); ^13^C-NMR (CDCl_3_) δ: 209.4 (C-20), 169.3 (C-3), 140.0, 103.0, 63.5, 56.6, 47.8, 44.0, 38.5, 34.3, 31.6, 31.5(2), 29.6, 28.5, 24.4, 22.8, 21.0, 18.7, 13.3; GC-MS (EI) *m/z*: 315 [M]^+^, 300 [M-NH]^+^; IR, cm^−1^: 3198 (N-H), 1702 (20-C=O), 1658 (3-C=O).

#### 3.2.3. 4-Aza-5-pregnene-3,20-dione-20-oxime (**4**)

4-Aza-5-pregnene-3,20-dione (**3**, 200 mg, 0.63 mmol) was dissolved in ethanol (10 mL) and NH_2_OH·HCl (66 mg, 0.95 mmol), triethylamine (1 mL) were added at room temperature and the reaction mixture was refluxed for 12 h. The reaction mixture was cooled down to room temperature, concentrated and added with H_2_O and extracted with dichloromethane. The combined organic layers were dried with anhydrous MgSO_4_, filtered, and concentrated to crude oily product which was purified by column chromatography (ethyl acetate-*n*-hexane = 1:3) to give **4** as a white solid. Yield: 147 mg (71%), m.p.: 208–210 °C; ^1^H-NMR (CDCl_3_) δ: 7.36 (1H, s, NH), 4.82 (1H, t, *J* = 3.4 Hz, H-6), 1.87 (3H, s, 21-CH_3_), 1.10 (3H, s, 19-CH_3_), 0.67 (3H, s, 18-CH_3_); ^13^C-NMR (CDCl_3_) δ: 169.6 (C-3), 158.9 (C=NOH), 140.2 (C-5), 103.3 (C-6), 56.9, 56.1, 48.2, 44.1, 38.6, 34.6, 32.0, 31.8, 29.8, 28.7, 24.3, 23.3, 21.1, 19.0, 15.4, 13.5; GC-MS (EI) *m/z*: 330 [M]^+^, 315 [M-NH]^+^; IR, cm^−1^: 3575 (OH), 3200 (N-H), 1668 (C=N), 1660 (3-C=O), 953 (N-O).

#### 3.2.4. 4-Aza-pregnane-3,20-dione (**5**)

10% Pd-C (180 mg) was added to a solution of 4-aza-5-pregnene-3,20-dione (**3**, 300 mg 0.95 mmol) in 95% ethanol (100 mL) and the mixture shaken in a Parr hydrogenation apparatus (50 psi) at 45 °C for 5 h. The reaction mixture was filtered on a Celite pad and the filtrate was evaporated under reduced pressure to form a white precipitate. The crude product was purified by silica gel column chromatography (methanol-dichloromethane = 1:49) to give **5** as a white solid. Yield: 244 mg (81%), m.p.: 275–277 °C; ^1^H-NMR (CDCl_3_) δ: 5.69 (1H, s, NH), 3.06 (1H, dd, *J*_1 _= 4.0 Hz, *J*_2 _= 12.0 Hz, H-5), 2.53 (1H, t, *J* = 8.8 Hz, H-17), 2.12 (3H, s, 21-CH_3_), 0.90 (3H, s, 19-CH_3_), 0.63 (3H, s, 18-CH_3_); ^13^C-NMR (CDCl_3_) δ: 209.4 (C-20), 172.1 (C-3), 63.6, 60.7, 56.1, 51.1, 44.3, 38.7, 35.8, 35.0, 33.4, 31.5, 29.5, 28.6, 27.4, 24.3, 22.8, 21.1, 13.5, 11.3; GC-MS (EI) *m/z*: 317 [M]^+^, 302 [M-NH]^+^; IR, cm^−1^: 3578 (OH), 3195 (N-H), 1705 (20-C=O), 1668 (3-C=O), 1675 (C=N), 954 (N-O).

#### 3.2.5. 4-Aza-pregnane-3,20-dione-20-oxime (**6**)

4-Aza-pregnane-3,20-dione (**5**, 100 mg, 0.32 mmol) was dissolved in ethanol (10 mL) and NH_2_OH·HCl (48 mg, 0.47 mmol), triethylamine (0.5 mL) were added at room temperature and the reaction mixture was refluxed for 12 h. The reaction mixture was cooled down to room temperature, concentrated and added with H_2_O and extracted with dichloromethane. The combined organic layers were dried with anhydrous MgSO_4_, filtered, and concentrated to crude oily product which was purified by column chromatography (ethyl acetate-*n*-hexane = 1:3) to give **6** as a white solid. Yield: 83 mg (78%), m.p.: 194–196 °C; ^1^H-NMR (CDCl_3_) δ: 7.68 (1H, s, NH), 5.54 (1H, br s, NOH), 1.87 (3H, s, 21-CH_3_), 0.91 (3H, s, 19-CH_3_), 0.64 (3H, s, 18-CH_3_); ^13^C-NMR (CDCl_3_) δ: 172.1 (C-3), 158.8 (C=NOH), 60.8, 56.7, 55.4, 51.3, 44.1, 38.7, 35.9, 35.3, 33.4, 29.5, 28.6, 27.5, 24.1, 23.0, 21.2, 15.1, 13.4, 11.4; GC-Mass (EI) *m/z*: 332 [M]^+^, 317 [M-NH]^+^; IR, cm^−1^: 3565 (OH), 3221 (N-H), 1665 (3-C=O).

#### 3.2.6. 4-Aza-1-pregnene-3,20-dione (**7**)

To a solution of 4-aza-pregnane-3,20-dione (**5**, 100 mg, 0.31 mmol) in 1,4-dioxane (10 mL) was added 2,3-dichloro-5,6-dicyano-1,4-benzoquinone (DDQ, 113 mg, 0.50 mmol) and refluxed. Bis (trimethylsilyl)trifluoroacetamide (BSTFA, 0.3 mL) was added dropwise to the refluxing reaction mixture and reacted for an additional 5 h at same temperature. The reaction mixture was filtered, washed with 1% Na_2_SO_3_ (aq.), 5% Na_2_SO_3_ (aq.) and H_2_O and extracted with dichloromethane. The organic phase was dried with anhydrous MgSO_4_, filtered, and concentrated in vacuum to afford the crude residue product which was purified by silica gel column chromatography (ethyl acetate-*n*-hexane = 1:3) to give **7** as a white solid. Yield: 64 mg (66%), m.p.: 174–176 °C; ^1^H-NMR (CDCl_3_) δ: 6.80 (1H, d, *J* = 10.0 Hz, H-1), 5.82 (1H, dd, *J*_1_ = 2.0 Hz, *J*_2 _= 10.0 Hz, H-2), 5.48 (1H, s, NH), 3.34 (1H, dd, *J*_1_ = 7.2 Hz, *J*_2 _= 9.2 Hz, H-5), 2.54 (1H, t, *J* = 9.0 Hz, H-17), 2.13 (3H, s, 21-CH_3_), 0.98 (3H, s, 19-CH_3_), 0.64 (3H, s, 18-CH_3_); ^13^C-NMR (CDCl_3_) δ: 209.2 (C-20), 166.5 (C-3), 150.9 (C-1), 123.0 (C-2), 63.5, 59.7, 56.1. 47.5, 44.3 39.5, 38.7, 35.3, 31.5, 29.4, 26.0, 24.3, 22.9, 21.3, 13.6, 12.0; GC-MS (EI) *m/z*: 315 [M]^+^, 300 [M-NH]^+^; IR, cm^−1^: 3190 (N-H), 1665 (3-C=O).

#### 3.2.7. 4-Aza-1-pregnene-3,20-dione-20-oxime (**8**)

4-Aza-1-pregnene-3,20-dione (**7**, 100 mg, 0.32 mmol) was dissolved in ethanol (10 mL) and NH_2_OH·HCl (48 mg, 0.48 mmol), triethylamine (0.5 mL) were added at room temperature and the reaction mixture was refluxed for 12 h. The reaction mixture was cooled down to room temperature, concentrated and added with H_2_O and extracted with dichloromethane. The combined organic layers were dried with anhydrous MgSO_4_, filtered, and concentrated to crude oily product which was purified by column chromatography (ethyl acetate-*n*-hexane = 1:3) to give **8** as a white solid. Yield: 78 mg (74%), m.p.: 235–237 °C; ^1^H-NMR (CDCl_3_) δ: 6.78 (1H, d, *J* = 11.0 Hz, H-1), 5.67 (1H, dd, *J*_1_ = 2.2 Hz, *J*_2_ = 11.0 Hz, H-2), 5.48 (1H, s, NH), 3.34 (1H, dd, *J*_1_ = 7.2 Hz, *J*_2 _= 9.2 Hz, H-5), 2.54 (1H, t, *J* = 9.0 Hz, H-17), 2.13 (3H, s, 21-CH_3_), 0.98 (3H, s, 19-CH_3_), 0.64 (3H, s, 18-CH_3_); ^13^C-NMR (MHz, CDCl_3_) δ: 168.8 (C-3), 156.3 (C=NOH), 151.8 (C-1), 124.5 (C-2), 62.5, 59.7, 55.1. 46.5, 44.3 39.5, 36.7, 34.3, 30.2, 28.4, 24.0, 22.3, 20.9, 21.3, 18.3, 14.0; GC-MS (EI) *m/z*: 330 [M]^+^; IR, cm^−1^: 3576 (OH), 3123 (N-H), 1673 (C=N), 1660 (3-C=O), 954 (N-O).

#### 3.2.8. 1,4,6-Pregnatriene-3,20-dione (**9**)

To a solution of 5-pregnen-3β-ol-20-one (pregnenolone, 3.0 g, 9.46 mmol) in 1,4-dioxane (40 mL), 2,3-dichloro-5,6-dicyano-1,4-benzoquinone (DDQ, 8.6 g, 37.90 mmol) was added and refluxed for 4 h. Yield : 1.3 g (44%), m.p.: 144–146 °C (145–147 °C) [29].

#### 3.2.9. 1α,2α-Epoxypregna-4,6-diene-3,20-dione (**10**)

1,4,6-Pregnatriene-3,20-dione (**9**, 500 mg, 1.61 mmol) was dissolved in methanol (25 mL) and 30% hydrogen peroxide (4.5 mL) and 5% NaOH-MeOH (1.2 mL) were added and stirred at room temperature for 4 h. Yield: 360 mg (68%), m.p.: 183–185 °C (184–187 °C) [29].

#### 3.2.10. 1α,2α-Epoxypregna-4,6-diene-3,20-dione-20-oxime (**11**) and 1α,2α-epoxypregna-4,6-diene-3,20-dioxime (**12**)

1α,2α-Epoxypregna-4,6-diene-3,20-dione (**10**, 100 mg, 0.31 mmol) was dissolved in ethanol (10 mL) and NH_2_OH·HCl (32.3 mg, 0.47 mmol), triethylamine (0.5 mL) were added at room temperature and the reaction mixture was refluxed for 6 h. The reaction mixture was cooled down to room temperature, concentrated and added with H_2_O and extracted with dichloromethane. The combined organic layers were dried with anhydrous MgSO_4_, filtered, and concentrated to crude oily product which was purified by column chromatography (ethyl acetate-*n*-hexane = 1:1) to give two compounds **11** and **12** as a white solid. 

Compound **11**: Yield: 41 mg (39%), m.p.: 228–230 °C; ^1^H-NMR (DMSO-*d_6_*) δ: 10.28 (1H, s, 20-NOH), 6.10 (1H, d, *J* = 9.2 Hz, H-7), 6.01 (1H, d, *J* = 9.2 Hz, H-6), 5.87 (1H, s, H-4), 3.60 (1H, s, H-2), 3.41 (1H, s, H-1), 1.71 (3H, s, 21-CH_3_), 1.05 (3H, s, 19-CH_3_), 0.68 (3H, s, 18-CH_3_); ^13^C-NMR (CDCl_3_) δ: 189.4 (C-3), 156.2 (C-5), 148.5 (C-20), 134.7 (C-7), 128.1 (C-6), 117.3 (C-4), 58.0 (C-1), 55.9 (C-2), 53.1, 52.9, 45.3, 43.3, 37.5, 37.4, 36.4, 22.8, 22.0, 21.5, 18.2, 15.8, 13.6; GC-MS (EI) *m/z*: 341 [M]^+^; IR, cm^−1^: 3569 (OH), 1673 (C=N), 1661 (3-C=O), 950 (N-O). 

Compound **12**: Yield : 23 mg (21%), m.p.: >230 °C (dec.); ^1^H-NMR (DMSO-*d_6_*) δ : 11.34 (1H, s, 3-NOH), 10.39 (1H, s, 20-NOH), 6.15 (1H, s, H-4), 6.11 (1H, d, *J* = 9.6 Hz, H-6), 5.87 (1H, d, *J* = 9.6 Hz, H-7), 3.63 (1H, s, H-2), 3.50 (1H, s, H-1), 1.78 (3H, s, 21-CH_3_), 1.01 (3H, s, 19-CH_3_), 0.64 (3H, s, 18-CH_3_), ^13^C-NMR (CDCl_3_) δ: 155.6 (C-5), 149.5 (C-20), 147.3 (C-3), 135.9 (C-7), 129.0 (C-6), 109.4 (C-4), 58.1 (C-1), 56.4 (C-2), 53.2, 52.5, 46.0, 44.3, 38.5, 38.4, 37.4, 23.9, 23.2, 21.3, 18.2, 15.8, 13.6; GC-MS (EI) *m/z*: 356 [M]^+^; IR, cm^−1^: 3558 (OH), 1670 (C=N), 953 (N-O).

#### 3.2.11. 6α,7α-Epoxypregna-1,4-diene-3,20-dione (**13**)

To a solution of 1,4,6-pregnatriene-3,20-dione (**9**, 200 mg, 0.64 mmol) in chloroform (20 mL), *m*-chloroperoxybenzoic acid (276.1 mg, 1.60 mmol) was added and stirred at room temperature for 24 h. Water was added to the reaction mixture which was washed with FeSO_4_ (aq.), H_2_O and extracted with dichloromethane. The organic layer was washed with H_2_O and dried with anhydrous MgSO_4_, filtered, concentrated to crude precipitate which was purified by column chromatography (ethyl acetate-*n*-hexane = 1:3) to give **10** as a pure white solid. Yield: 98 mg (48%), m.p.: 206–208 °C; ^1^H-NMR (CDCl_3_) δ: 7.01 (1H, d, *J =* 10.4 Hz, H-1), 6.49 (1H, s, H-4), 6.24 (1H, dd, *J*_1_ = 1.8 Hz, *J*_2 _= 10.2 Hz, H-2), 3.65 (1H, d, *J* = 3.2 Hz, H-7), 3.37 (1H, d, *J* = 3.2 Hz, H-6), 2.59 (1H, t, *J* = 9.4 Hz, H-17), 2.14 (3H, s, 21-CH_3_), 1.21 (3H, s, 19-CH_3_), 0.73 (3H, s, 18-CH_3_); ^13^C-NMR (CDCl_3_) δ: 208.9 (C-20), 185.2 (C-3), 159.6 (C-5), 153.1 (C-1), 131.2 (C-2), 127.9 (C-4), 62.9 (C-7), 53.8 (C-6), 51.7, 51.3, 44.3, 41.0, 38.2, 35.3, 31.5, 23.7, 23.0, 21.9, 20.6, 13.3; GC-MS (EI) *m/z*: 326 (M^+^), 308 (M^+^-H_2_O); IR, cm^−1^: 1671 (20-C=O), 1666 (3-C=O).

#### 3.2.12. 6α,7α-Epoxypregna-1,4-diene-3,20-dione-20-oxime (**14**)

6α,7α-Epoxypregna-1,4-diene-3,20-dione (**10**, 100 mg, 0.31 mmol) was dissolved in ethanol (10 mL) and NH_2_OH·HCl (32.3 mg, 0.47 mmol), and triethylamine (0.5 mL) were added at room temperature and the reaction mixture was refluxed for 6 h. After cooling down to room temperature, the reaction mixture was concentrated, H_2_O added and the mixture extracted with dichloromethane. The combined organic layers were dried with anhydrous MgSO_4_, filtered, and concentrated to crude oily product which was purified by column chromatography (ethyl acetate-*n*-hexane = 1:3) to give **14** as a white solid. Yield: 67 mg (63%), m.p.: 245–247 °C; ^1^H-NMR (DMSO-*d_6_*) δ: 10.29 (1H, s, 20-NOH), 7.15 (1H, d, *J* = 10 Hz, H-1), 6.23 (1H, s, H-4), 6.07 (1H, d, *J* = 10.0 Hz, H-2), 3.70 (1H, *J* = 3.2 Hz, H-7), 3.34 (1H, *J* = 3.2 Hz, H-6), 1.65 (3H, s, 21-CH_3_), 1.29 (3H, s, 19-CH_3_), 0.56 (3H, s, 18-CH_3_); ^13^C-NMR (CDCl_3_) δ: 185.4 (C-3), 160.4 (C-5), 157.7 (C=NOH), 155.4(C-1), 130.7 (C-2), 126.3 (C-4), 72.6 (C-7), 63.3 (C-6), 56.5, 49.1, 43.4, 43.3, 41.9, 38.1, 34.3, 23.6, 23.0, 22.7, 21.9, 15.6, 13.4; GC-MS (EI) *m/z*: 341 [M]^+^; IR, cm^−1^: 3567 (OH), 1673 (C=N), 945 (N-O).

### 3.3. Testosterone 5α-Reductase Assay

The enzyme suspension of testosterone 5α-reductase was prepared from the homogenate of the ventral prostate of male Sprague-Dawley rats using the methods previously reported [32]. Testosterone 5α-reductase inhibition was measured according to the methods previously reported [32]. A brief description follows: Sprague-Dawley male rats 7-8 weeks old were sacrificed by diethyl ether, and the ventral prostates were excised and minced with a pair of scissors. The prostates were homogenized with a glass-glass homogenizer in medium A (0.32 M sucrose, 0.1 mM dithiothreitol and 20 mM sodium phosphate, pH 6.5) to about 20% (w/v). The homogenate was filtered over 8 layers of surgical gauze, and centrifuged at 3,000 × *g* for 15 min. The resulting pellets were resuspended in medium A to about 25% (w/v) by triturating the suspension sequentially through an 18-gauge and then a 20-gauge needle. The assay mixture containing the test sample in ethanol (10 μL), reaction solution [525 μL, 1 mM dithiothreitol, 40 mM sodium phosphate (pH 6.5), 50 µM NADPH and 2.2 mM [1,2,6,7-^3^H]testosterone, 3.15-3.89 TBq/mmol, PerkinElmer (Waltham, MA, USA)] and the enzyme suspension prepared above (40 μL), was incubated at 37°C for 1 h. After extraction with ethyl acetate (1 mL), a sample of the ethyl acetate phase (50 μL) was chromatographed on a silica plastic sheet (Kieselgel 60 F_254_, Merck KGaA (Darmstadt, Germany)) using the developing solvent system ethyl acetate-cyclohexane (1:1). Each of the testosterone and 5α-dihydrotestosterone areas was cut from the sheet. The ^3^H radioactivity of each fragment was counted in 5 mL of Aquasol-2 (PerkinElmer) with a liquid scintillation counter [LS 6500, Beckman Coulter (Brea, CA, USA)]. Testosterone 5α-reductase inhibitory activity was calculated as follows:


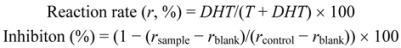


where *DHT* and *T* are the ^3^H radioactivity recovered in the areas of 5α-dihydrotestosterone and testosterone, respectively. *r*_sample_ and *r*_control_ are reaction rates of the test sample and the enzyme control in which reaction rates are measured in the presence and absence of test sample, respectively, and *r*_blank_ is reaction rate of the substrate blank in which reaction rate is measured in the absence of test sample and enzyme suspension. IC_50_ was calculated from the inhibitory activity values at several concentrations using linear regression analysis. Each enzyme reaction was carried out in duplicate.

## 4. Conclusions

In this paper we have described the synthesis of novel 20-hydroxyimino substituted C21 steroids having additional double bonds in the A and/or B ring and 20-hydroxyimino-4-azasteroid derivatives and their evaluation as inhibitors against 5α-reductase. All synthesized compound showed less activity than finasteride. While the hydroxyimino group at the 20-position of a C_21_ steroid skeleton enhanced the inhibitory activity in some 4-azasteroid derivatives (compounds **4**, **6** and **8**), the presence of an additional double bond and an epoxy group at the 1,2-position in the molecule might significantly reduce the inhibitory activity regardless of steroid 20-one or 20-oxime analogs. 
